# Pregnancy‐Associated Small Cell Carcinoma of the Ovary, Hypercalcaemic Type Initially Misdiagnosed as Granulosa Cell Tumour: Diagnostic Pitfalls, Treatment Challenges, and Molecular Confirmation by Next‐Generation Sequencing

**DOI:** 10.1002/cnr2.70628

**Published:** 2026-07-16

**Authors:** Mahendra Kumar, Lopamundra Kakoti, Debabrata Barmon, Upasana Baruah, Dimpy Begum, P. S. Roy, Karthik Chandra Bassetty, Deepali Mittal, Bharati Seth

**Affiliations:** ^1^ Department of Gynaecological Oncology Dr B Borooah Cancer Institute Guwahati Assam India; ^2^ Department of Oncopathology Dr Bhubaneshwar Borooah Cancer Institute Guwahati Assam India; ^3^ Department of Medical Oncology Dr Bhubaneshwar Borooah Cancer Institute Guwahati Assam India; ^4^ Department of Gynaecological Oncology Dr Bhubaneshwar Borooah Cancer Institute Guwahati Assam India

**Keywords:** Hypercalcaemic ovarian cancer, rare ovarian cancer, small cell ovarian cancer

## Abstract

**Background:**

Small cell carcinoma of the ovary, hypercalcaemic type (SCCOHT), is an exceptionally rare and highly aggressive ovarian malignancy affecting predominantly young women. Its overlapping morphology with other poorly differentiated ovarian tumours and lack of specific early markers frequently lead to diagnostic delay. Universal loss of SMARCA4 expression defines the disease and has critical diagnostic and therapeutic implications.

**Case Presentation:**

We describe a young woman diagnosed with an ovarian malignancy during pregnancy (12.6 weeks), initially interpreted as a granulosa cell tumour based on histomorphology. She underwent surgical management (initial surgery during pregnancy at 16.2 weeks, hysterectomy at 39 weeks) followed by platinum‐based chemotherapy and achieved an initial complete radiologic response. However, the disease recurred rapidly with extensive peritoneal, visceral, skeletal, and cutaneous metastases, including scar‐site involvement. Recurrent tumour histology showed an undifferentiated phenotype with a non‐contributory immunohistochemical profile, resulting in significant diagnostic ambiguity. A definitive diagnosis was established by next‐generation sequencing, which identified a pathogenic SMARCA4 alteration, confirming SCCOHT. Despite aggressive multimodal therapy, the disease progressed relentlessly with rapid clinical deterioration.

**Conclusion:**

This case underscores the profound diagnostic challenges posed by SCCOHT, particularly when presenting during pregnancy and mimicking sex cord–stromal tumours. It highlights the limitations of morphology and immunohistochemistry alone in poorly differentiated ovarian neoplasms and reinforces the indispensable role of SMARCA4 testing and molecular profiling for accurate diagnosis. Early recognition is essential to guide aggressive multimodal therapy, genetic counselling, and consideration of emerging targeted strategies. This report adds to the limited literature on SCCOHT and emphasises the need for heightened clinical suspicion and precision diagnostics in young women with rapidly progressive ovarian malignancies.

## 1 Introduction

Small‐cell carcinoma of the ovary, hypercalcaemic type (SCCOHT), is an exceptionally rare but highly aggressive ovarian malignancy, accounting for less than 0.1% of all ovarian cancers [1, 2, 3, 4] First described by Dickersin and colleagues in 1982, SCCOHT predominantly affects adolescents and young women, with a median age at diagnosis of approximately 24 years [1, 2, 3, 4] Clinically, patients commonly present with rapidly progressive abdominal pain, abdominal distension, pelvic mass, or nonspecific gastrointestinal symptoms. Hypercalcaemia is observed in approximately two‐thirds of cases and may manifest as nausea, vomiting, constipation, fatigue, polyuria, altered mental status, or may remain asymptomatic and be detected only on laboratory evaluation [1, 2, 3, 4] The disease is characterised by aggressive biological behaviour, frequent advanced‐stage presentation, early recurrence, and poor long‐term survival [1, 2, 5].

Preoperative diagnosis of SCCOHT remains particularly challenging because there are no pathognomonic clinical, radiological, or serum tumour marker findings. Imaging studies typically demonstrate a large unilateral ovarian mass, often exceeding 10 cm in diameter, with heterogeneous solid and cystic components, areas of haemorrhage, necrosis, and variable contrast enhancement on computed tomography (CT) or magnetic resonance imaging (MRI) [1, 5, 6] Serum tumour markers such as CA‐125 may be elevated but are nonspecific, while germ‐cell tumour markers are generally negative. Consequently, SCCOHT is rarely diagnosed preoperatively, and the diagnosis often relies on postoperative histopathological examination supplemented by immunohistochemistry and molecular testing [5, 7, 8, 9].

Pathologically, SCCOHT is characterised by undifferentiated small‐to‐medium cells with rhabdoid features, and a highly characteristic molecular feature is the loss of SMARCA4 (BRG1) expression, which serves as a key diagnostic marker and helps distinguish it from other ovarian malignancies. These features create substantial diagnostic overlap with granulosa cell tumours, germ‐cell tumours, neuroendocrine carcinomas, lymphomas, Ewing sarcoma, and other small round blue cell tumours of the ovary [5, 8, 9]. Immunohistochemistry is often essential in narrowing the differential diagnosis; however, the immunophenotype can be variable and occasionally non‐specific. The most characteristic diagnostic feature is loss of SMARCA4 (BRG1) protein expression, which is observed in the vast majority of SCCOHT cases and serves as a highly sensitive and specific diagnostic marker [5, 7, 8, 9].

Genetically, SCCOHT is driven almost universally by inactivation of the SMARCA4 gene, placing it within the spectrum of SWI/SNF‐deficient rhabdoid tumours [1, 5, 7, 8, 9] Germline SMARCA4 mutations have been identified in up to 40% of patients, carrying important implications for hereditary cancer predisposition, genetic counselling, and family screening [1, 7, 8] Advances in molecular profiling have significantly improved diagnostic accuracy and have stimulated interest in targeted therapeutic strategies, including EZH2 inhibition, immune checkpoint blockade, and synthetic lethal approaches targeting SWI/SNF‐deficient tumours, although these treatments remain largely investigational [9, 10, 11, 12].

Management of SCCOHT remains challenging because of its rarity and the absence of standardised treatment guidelines. Current evidence supports an aggressive multimodal approach incorporating maximal cytoreductive surgery, platinum‐based chemotherapy, and selected use of radiotherapy [1, 2, 6, 13]. High‐dose chemotherapy followed by autologous stem‐cell transplantation has demonstrated encouraging outcomes in selected patients, although overall prognosis remains poor, with most patients experiencing recurrence within 12–18 months and advanced‐stage five‐year survival rates remaining below 10% [1, 2, 6, 7, 13]. Emerging targeted and epigenetic therapies may improve future outcomes but require further clinical validation [10, 11, 12, 14, 15].

Given the tumour's rarity, diagnostic complexity, and aggressive clinical course, detailed case reports remain invaluable for improving recognition, refining diagnostic algorithms, and guiding personalised management strategies. The present case highlights the challenges of diagnosing SCCOHT during pregnancy, its initial misclassification as a granulosa cell tumour, and the pivotal role of molecular testing in establishing the final diagnosis.

## 2 Case Report

A 39‐year‐old P2L2 woman was incidentally diagnosed with a left adnexal mass during a routine antenatal ultrasound examination at 12.6 weeks of gestation in her second pregnancy in May 2023. She was asymptomatic, with no significant past medical or family history, and reported no abdominal pain, abdominal distension, gastrointestinal or urinary symptoms, weight loss, or features suggestive of hypercalcaemia. Tumour markers were performed, and all were within normal limits except for CA‐125, which was elevated at 222 IU/mL.

In May 2023, at 16.2 weeks of gestation, she underwent exploratory laparotomy with left ovarian cystectomy at a nursing home. Histopathological examination suggested a high‐grade juvenile granulosa cell tumour. The tumour measured approximately 12 × 10 cm, and no comprehensive staging procedure was performed at the time of surgery. A second laparotomy at 29 weeks of gestation in August 2023 at the same nursing home resulted in left oophorectomy, which again was reported as a juvenile granulosa cell tumour. No adjuvant treatment was administered during pregnancy because of the advanced gestational age.

At term (39 weeks), she underwent a caesarean hysterectomy with right oophorectomy at the same institution. Intraoperatively, focal tumour deposits were identified over the uterine serosa and rectus sheath. Histopathological examination demonstrated focal surface deposits and a rectus sheath nodule positive for granulosa cell tumour, suggesting metastatic disease.

**FIGURE 1:**
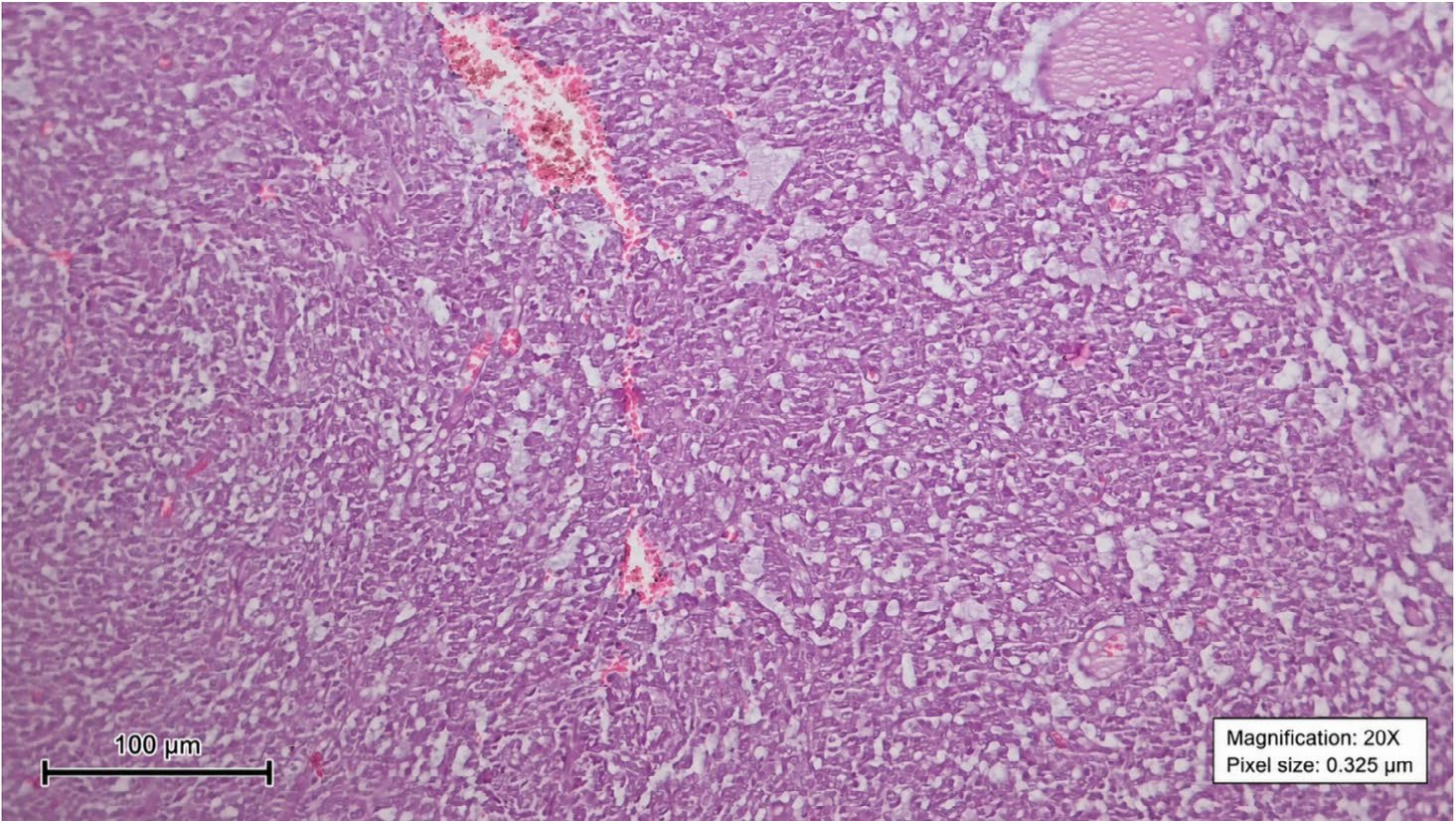
Haematoxylin and eosin (H&E)‐stained section of the ovarian tumour (post oophorectomy) showing a highly cellular malignant neoplasm composed of diffuse sheets and nests of poorly differentiated cells separated by delicate fibrovascular septae (original Magnification 20×; pixel size 0.325 μm). The tumour demonstrates marked architectural disorganisation with areas of necrosis and haemorrhage. The morphology lacks the characteristic nuclear grooves and microfollicular architecture typically seen in granulosa cell tumours, contributing to the diagnostic uncertainty encountered during initial pathological evaluation.

**FIGURE 2:**
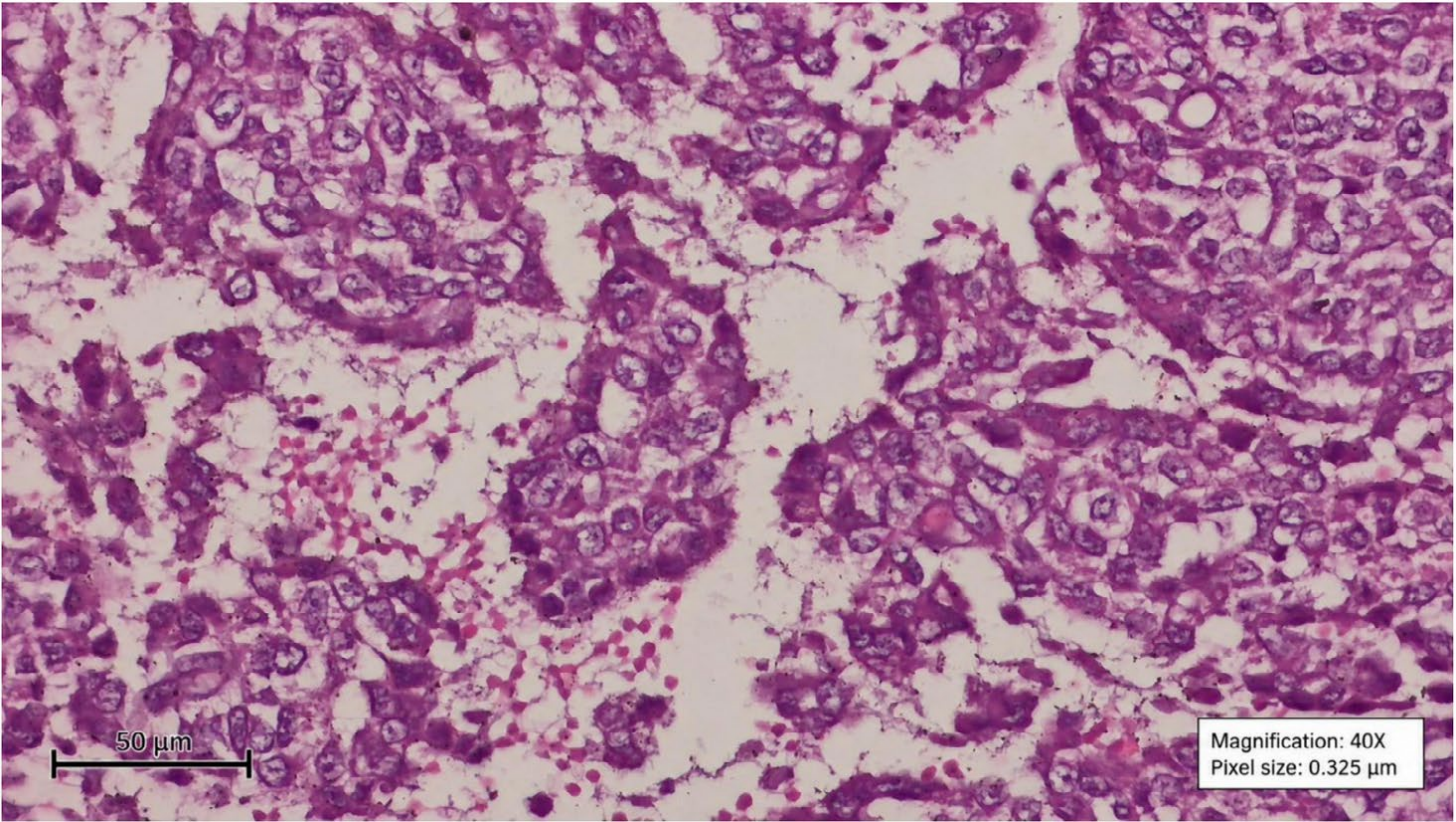
High‐power H&E photomicrograph demonstrating sheets of undifferentiated round‐to‐oval malignant cells with a high nuclear‐to‐cytoplasmic ratio, vesicular chromatin, conspicuous nucleoli, and brisk mitotic activity (original magnification 40×; pixel size 0.325 μm). Tumour cells show marked nuclear pleomorphism and focal rhabdoid morphology with abundant eosinophilic cytoplasm. The absence of definitive sex‐cord stromal differentiation and the poorly differentiated appearance raised a broad differential diagnosis, including granulosa cell tumour, undifferentiated sarcoma, Ewing sarcoma, and small‐cell carcinoma of the ovary, ultimately necessitating molecular testing for definitive classification.

Following delivery, the patient was referred to Dr. Bhubaneswar Borooah Cancer Institute (BBCI), Guwahati, for further management. Review of all outside histopathology slides revised the diagnosis to adult‐type granulosa cell tumour (Figures 1 and 2). Immunohistochemistry demonstrated an unusual profile with negativity for calretinin, inhibin, SF1, CD117, and NKX2.2, while the Ki‐67 proliferation index was approximately 70%, raising concern regarding the original diagnosis.

Baseline contrast‐enhanced computed tomography (CECT) demonstrated multiple peritoneal nodules involving the pelvis, omentum, mesentery, abdominal wall, and rectus sheath, consistent with disseminated intra‐abdominal disease.

After multidisciplinary tumour board discussion, the patient received three cycles of bleomycin, etoposide, and cisplatin (BEP) chemotherapy. Interval imaging demonstrated a partial radiological response according to RECIST criteria, following which she underwent interval cytoreductive surgery at Dr. Bhubaneswar Borooah Cancer Institute, Guwahati. The procedure included complete excision of all visible residual tumour deposits, omentectomy, excision of peritoneal deposits, and abdominal wall tumour excision, resulting in complete macroscopic cytoreduction (PCI = 12, CC‐0 resection).

Histopathological examination of the interval debulking specimen revealed a poorly differentiated malignant neoplasm. Based on morphology, the differential diagnosis included endometrial stromal sarcoma and undifferentiated sarcoma. An extensive immunohistochemical panel was subsequently performed; however, the findings remained inconclusive and failed to support a definitive diagnosis of sarcoma.

The patient subsequently received three additional cycles of etoposide and cisplatin (EP) chemotherapy. Post‐treatment assessment in July 2024 demonstrated a complete clinical, biochemical, and radiological response. Complete response was defined by the absence of clinical evidence of disease, normalisation of serum tumour markers, and contrast‐enhanced CT demonstrating complete resolution of all previously identified lesions without residual measurable disease (Table 1).

**TABLE 1:** Clinicopathological summary of surgeries, biopsies, and immunohistochemical findings.

No	Date	Surgery performed	Weeks of gestation	HPE	IHC
1.	May 2023	Left ovarian cystectomy	16.2 Weeks	High grade Juvenile granulosa cell tumour	Not done
2.	August 2023	Left oophorectomy	29 weeks	High grade Juvenile granulosa cell tumour	Not done
3.	October 2023	Caesarean hysterectomy with right oophorectomy	39 weeks	High‐grade adult‐type granulosa cell tumour	Calretinin, Inhibin, CD117, NKX2.2, Ki67–70%—All Negative.
4.	March 2024	Debulking surgery		Sarcoma—Endometrial stromal sarcoma/Undifferentiated Sarcoma	CK, Vimentin, Inhibin, CD45, CD99, NKX2.2, EMA, PAX8, SF1, cyclin D1, CD10 and AE1/AE3—All Negative
5.	September 2024	Abdominal wall biopsy		High‐grade undifferentiated malignant tumour	Nil

Abbreviations: AE1/AE3—Broad‐spectrum Cytokeratin Antibody Cocktail; CD—Cluster of Differentiation; CK—Cytokeratin; EMA—Epithelial Membrane Antigen; HPE—Histopathological Examination; IHC—Immunohistochemistry; Ki‐67—Ki‐67 Proliferation Index; NGS—Next‐Generation Sequencing; NKX2.2—NK2 Homeobox 2; PAX8—Paired Box Gene 8; SCCOHT—Small‐Cell Carcinoma of the Ovary, Hypercalcaemic Type; SF1—Steroidogenic Factor 1.

The patient remained under surveillance with scheduled follow‐up every three months. Approximately six months after completion of treatment, she presented with a progressively enlarging swelling over the previous abdominal scar. Core biopsy revealed a high‐grade undifferentiated malignant tumour. Contrast‐enhanced CT demonstrated a large recurrent pelvic mass associated with extensive metastatic disease involving the liver, spleen, retroperitoneal lymph nodes, skeletal system, and abdominal wall scar.

Repeat immunohistochemistry remained non‐contributory, demonstrating negativity for calretinin, inhibin, PAX8, CD45, and NKX2.2, with only faint CD99 expression. Because the morphology and immunophenotype remained inconclusive despite repeated pathological review, the case was discussed again in the multidisciplinary tumour board, and comprehensive molecular profiling was recommended.

**FIGURE 3:**
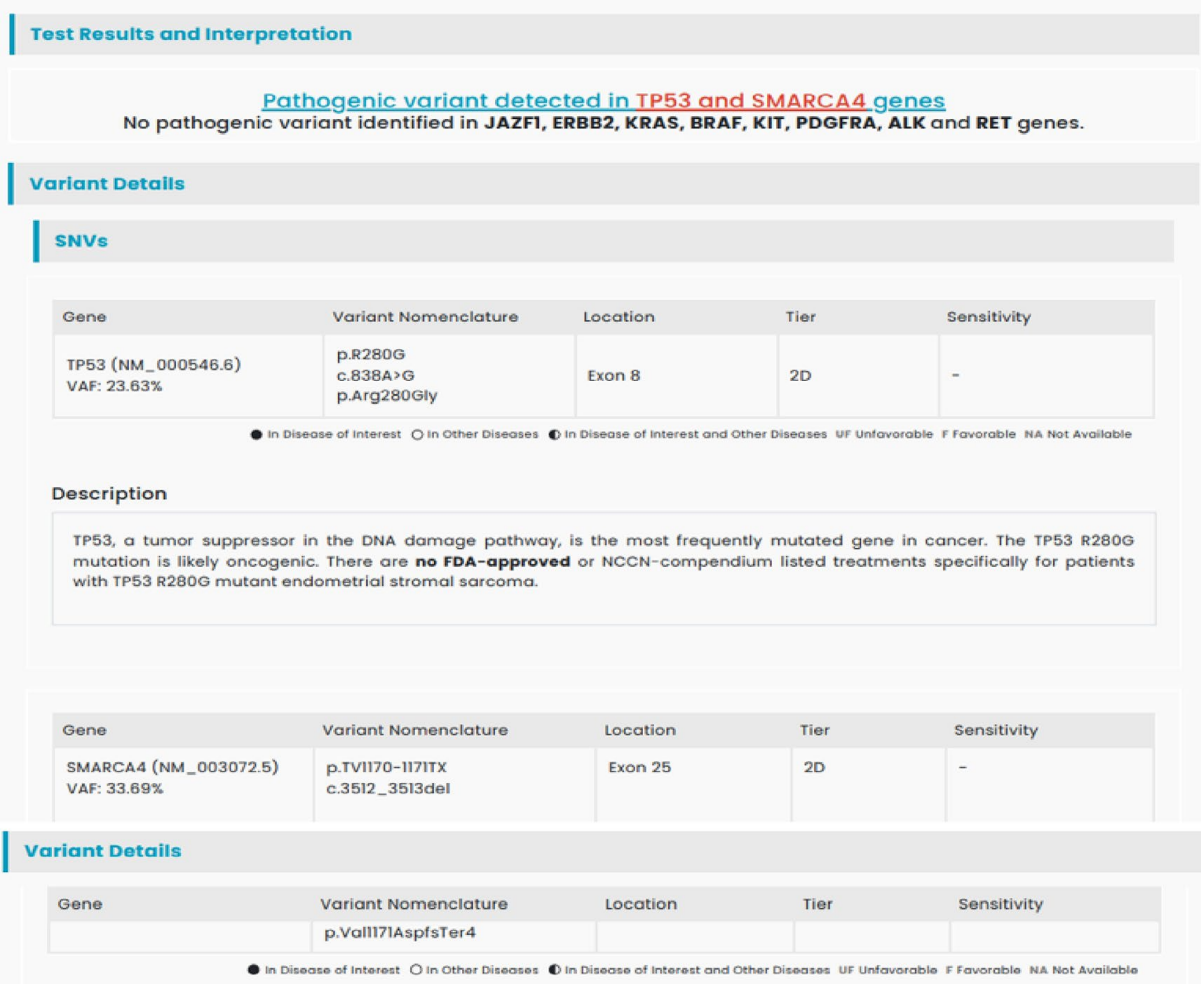
Next‐generation sequencing (NGS) analysis demonstrating pathogenic alterations in TP53 and SMARCA4 genes. The report identified a pathogenic TP53 mutation (p.Arg280Gly) and a truncating SMARCA4 alteration resulting in loss of normal SMARCA4 function. Detection of the SMARCA4 pathogenic variant provided the critical molecular evidence supporting the diagnosis of small‐cell carcinoma of the ovary, hypercalcaemic type (SCCOHT), a SMARCA4‐deficient ovarian malignancy. These findings resolved the diagnostic ambiguity created by the non‐specific histopathological and immunohistochemical features and established the final diagnosis.

Next‐generation sequencing (NGS) subsequently identified pathogenic alterations involving SMARCA4 together with TP53, establishing the definitive diagnosis of SMARCA4‐deficient small‐cell carcinoma of the ovary, hypercalcaemic type (SCCOHT). (Figure 3).

Following confirmation of the diagnosis, the patient was evaluated for further systemic therapy. However, because of rapidly progressive disease, poor performance status, and extensive metastatic burden, she was considered unsuitable for further intensive chemotherapy and was managed with best supportive and palliative care.

The disease progressed rapidly, with rupture of the abdominal wall scar, metastasis and progressive deterioration in general condition. Despite comprehensive supportive care, the patient succumbed to her illness in January 2025, approximately 20 months after her initial diagnosis and six months after achieving complete radiological response.

The patient, unfortunately, succumbed to disease progression during follow‐up. Written informed consent for publication was subsequently obtained from the patient's legally authorised next of kin (her husband). Consent specifically covered publication of anonymised clinical data, treatment details, histopathological images, molecular findings, and radiological figures. All potentially identifiable information has been removed, and every reasonable effort has been undertaken to preserve patient confidentiality. The signed consent document is retained by the authors and can be provided to the journal upon request.

## 3 Discussion

Small cell carcinoma of the ovary, hypercalcaemic type (SCCOHT), continues to pose one of the greatest diagnostic and therapeutic challenges in gynaecologic oncology [1, 2, 3]. Its rarity, extreme biological aggressiveness, and overlap with other small round cell tumours frequently result in delayed or missed diagnosis, especially when patients present with atypical clinical trajectories [1, 2, 3, 6]. The present case uniquely illustrates the diagnostic complexity of SCCOHT, particularly when superimposed on an initial diagnosis of granulosa cell tumour, and highlights the rapid evolution of disease despite aggressive multimodal therapy.

Our patient's course was particularly unusual because the malignancy was first identified during pregnancy, a period that can complicate diagnostic evaluation due to limitations in imaging, surgical decision‐making, and pathological interpretation. These challenges contributed to the initial diagnostic uncertainty in our case, where the tumour was initially interpreted as a granulosa cell tumour.

The histopathological overlap between SCCOHT and other ovarian neoplasms is well recognised. SCCOHT typically presents as an undifferentiated tumour composed predominantly of monomorphic small cells arranged in sheets and nests, often with variable follicle‐like spaces and, in some cases, an admixed large‐cell component [1, 5, 9]. The presence of these follicle‐like spaces can closely mimic the microfollicular pattern of granulosa cell tumour, thereby contributing to diagnostic confusion [1, 5, 9]. Furthermore, the poorly differentiated morphology and often non‐specific immunohistochemical profile of SCCOHT can make distinction from granulosa cell tumour, undifferentiated carcinoma, germ‐cell tumours, and other small round‐cell malignancies particularly challenging, especially when specialised markers such as BRG1 (SMARCA4) are not routinely available.

Due to its highly aggressive and poorly differentiated morphology, SCCOHT presents a broad differential diagnosis ranging from undifferentiated or dedifferentiated carcinoma to granulosa cell tumour to other small round cell malignancies, including small cell carcinoma and germ cell tumours, especially embryonal or dysgerminoma [1, 5, 9, 16]. In addition to its variable morphology, the often ambiguous or non‐specific immunohistochemical profile further compounds the diagnostic difficulty, even for experienced pathologists, especially when tissue is fragmented or partially necrotic—conditions frequently encountered in gravid patients [1, 5, 9, 16]. Moreover, the limited availability of specialised immunohistochemical markers such as BRG1 (SMARCA4) and INI1 (SMARCB1) in many centres further adds to the diagnostic challenge [5, 9, 16]. (Table 2).

**TABLE 2:** Showing a different rare case report of SCCOHT.

No	Study	Mean age	IHC performed	NGS performed	Management	Outcome
1.	Young et al. (1994) [17]	Median 24 (range 1–46)	Variable epithelial markers; diagnosis based on morphology; SMARCA4 was not known at that time	Not performed	Surgery ± chemotherapy ± radiotherap	Extremely poor prognosis; most advanced‐stage patients died within 2 years
2.	Harrison et al. (2006) [18]	Median 26	Limited IHC; nonspecific cytokeratin, EMA positivity	Not performed	Surgery + platinum‐based chemotherapy ± radiotherapy	5‐year OS ~10% for advanced disease
3.	Pautier et al. (2007) [13]	Median 29	Conventional IHC; no specific marker	Not performed	Intensive multimodal therapy incl. surgery, multi‐agent chemotherapy, radiotherapy, ±HDCT‐ASCT	Improved survival in patients receiving aggressive combined therapy
4.	Callegaro‐Filho et al. (2016) [19]	Median 30	Retrospective review; SMARCA4 loss recognised in later cases	Partial (retrospective)	Surgery + platinum/etoposide or ifosfamide‐based chemotherapy ± radiotherapy	Median OS ~14.9 months; complete cytoreduction is associated with better outcomes
5.	Jelinic et al. (2014) [8]	Mean ~25	Universal loss of SMARCA4 (BRG1) on IHC	Yes—recurrent SMARCA4 mutations	Variable; surgery + chemotherapy	Established SMARCA4 as a defining driver mutation
6.	Witkowski et al. (2014) [20]	Mean 26	Complete loss of SMARCA4 in all tested cases	Yes (germline + somatic)	Multimodal therapy	Germline SMARCA4 mutations in ~40%; confirmed hereditary risk
7.	Hasselblatt et al. (2014) [21]	Median 24	SMARCA4 loss; rhabdoid phenotype	Yes	Surgery + chemotherapy	Reinforced classification as a rhabdoid tumour

The false sense of histologic clarity in the early phase was followed by an aggressive peritoneal dissemination pattern, observed on imaging shortly after delivery. The patient's multidisciplinary management pathway, including BEP chemotherapy followed by interval debulking surgery and EP consolidation, reflects contemporary practice for high‐grade ovarian neoplasms of uncertain lineage [1, 2, 6] Her complete radiologic response post‐chemotherapy aligns with the transient chemosensitivity reported in SCCOHT and related rhabdoid tumours, which often respond initially yet relapse dramatically due to the rapid emergence of chemoresistant clones [2, 6, 7, 13].

The turning point in this case was the patient's recurrence with scar‐site metastasis in the setting of disseminated metastatic disease. Although scar‐site involvement may result from tumour implantation during prior surgical procedures, the concurrent presence of extensive metastatic deposits involving the liver, spleen, retroperitoneum, skeleton, and peritoneal cavity suggested highly aggressive tumour biology and widespread disease progression [22]. The recurrent tumour exhibited an undifferentiated morphology with a poorly informative immunohistochemical profile, further highlighting the diagnostic challenges associated with advanced SCCOHT [23, 24]. The recurrent tumour's phenotype—undifferentiated morphology with a poorly informative initial immunohistochemical panel—demonstrated the tumour's dedifferentiated state. The absence of inhibin, calretinin, and PAX8, as well as SF1 expression, effectively ruled out granulosa cell tumour, sex cord stromal neoplasms, and Müllerian high‐grade carcinomas, respectively [5, 9, 16] The faint CD99 positivity and vimentin/EMA reactivity raised possibilities including Ewing‐family sarcoma or synovial sarcoma, but the lack of lineage‐specific markers rendered the IHC profile inconclusive [5, 9, 16].

This diagnostic ambiguity reflects a well‐documented challenge: recurrent SCCOHT often loses residual differentiation pathways and may masquerade as undifferentiated sarcoma or high‐grade carcinoma, creating significant diagnostic uncertainty [1, 5, 9, 16] At this juncture, molecular testing becomes indispensable. The tumour's NGS revealed a pathogenic SMARCA4 alteration, which provided the decisive diagnostic clue [1, 5, 7, 8, 9] SMARCA4 loss is now recognised as the defining hallmark of SCCOHT, and its identification allowed precise classification, even in a tumour that was otherwise morphologically and immunophenotypically ambiguous [1, 5, 7, 8, 9].

This case, therefore, reinforces the essential role of molecular profiling in the evaluation of undifferentiated ovarian malignancies. Without NGS, the diagnosis would have remained unresolved, potentially leading to inappropriate therapy choices. Incorporating routine SMARCA4 immunohistochemistry and, when needed, genomic testing in high‐grade, small‐cell, or rhabdoid ovarian tumours is critical for achieving diagnostic accuracy [1, 5, 7, 8, 9, 16].

The clinical behaviour of the tumour in our patient—rapid relapse within months of achieving complete response, widespread metastases involving the liver, spleen, retroperitoneum, and skeletal system, and final catastrophic skin and soft tissue infiltration—is entirely consistent with the highly aggressive natural history of SCCOHT [1, 2, 3, 6, 7]. The disease often progresses fulminantly, with a median survival for advanced‐stage patients of less than 12–18 months despite aggressive multimodality therapy [1, 2, 6, 7, 13]. Our patient's tragic deterioration mirrors the patterns described in the literature and underscores the lethality of this tumour.

Therapeutically, the case highlights the limitations of current management. Although she received platinum‐based therapy, debulking surgery, and consolidation chemotherapy—the existing standard of care—treatment failed to induce durable disease control [1, 2, 6, 13] A limitation in the diagnostic workup was the unavailability of BRG1 (SMARCA4) immunohistochemistry at our institution (Dr Bhubaneswar Borooah Cancer Institute, Guwahati). Given that loss of BRG1 expression is a highly characteristic feature of SCCOHT, earlier access to this marker may have facilitated a more timely diagnosis. In the absence of BRG1 testing, the diagnosis remained challenging because of overlapping morphological features and a non‐specific immunophenotype. Ultimately, next‐generation sequencing demonstrating a pathogenic SMARCA4 alteration was instrumental in establishing the final diagnosis. High‐dose chemotherapy with autologous stem‐cell rescue, which has been associated with improved survival in select patients, could not be offered due to late diagnosis and rapidly declining performance status [1, 2, 13] The emerging role of EZH2 inhibitors, immune checkpoint blockade, and synthetic lethal therapies targeting SWI/SNF‐deficient tumours was not clinically accessible due to the advanced disease stage and the patient's deteriorating condition [10, 11, 12, 14, 15, 23, 24].

This case also underscores an important diagnostic teaching point: any ovarian tumour in a young woman that is poorly differentiated, rapidly progressive, and immunohistochemically ambiguous should trigger immediate testing for SMARCA4 deficiency [1, 5, 7, 8, 9, 25]. Early recognition is key, as delay hampers optimal planning for aggressive combined modality therapy and genetic counselling for possible germline SMARCA4 mutation [1, 6, 7, 8]. An ovarian tumour that is high‐grade, composed of small‐to‐medium undifferentiated cells, negative for sex cord–stromal and epithelial markers, and showing a ‘null’ immunophenotype should immediately prompt SMARCA4 (BRG1) testing to exclude SCCOHT [5, 9, 16].

Recent reports have highlighted the unique challenges associated with SCCOHT diagnosed during pregnancy, including delays in diagnosis, limitations in imaging and treatment options, and the aggressive clinical course of the disease. Similar to previously reported cases, our patient presented during pregnancy and subsequently experienced rapid disease progression despite multimodality treatment. However, unlike most reported cases in which the diagnosis was established on initial pathological assessment, our patient underwent multiple diagnostic revisions, including interpretations of juvenile granulosa cell tumour, adult granulosa cell tumour, and undifferentiated sarcoma, before molecular testing ultimately confirmed SCCOHT. This prolonged diagnostic evolution underscores the significant morphological and immunophenotypic overlap between SCCOHT and other ovarian malignancies.

Therefore, rather than representing a unique clinical entity, this case adds to the growing body of evidence demonstrating the diagnostic complexity of SCCOHT in pregnancy and highlights the critical role of molecular testing in resolving diagnostically challenging ovarian tumours. Increased awareness of this entity and early incorporation of SMARCA4‐directed testing may facilitate more timely diagnosis and appropriate management.

## 4 Conclusion

In conclusion, this case highlights the formidable challenges posed by SCCOHT across diagnosis, treatment, and prognosis. It reinforces the importance of integrating histopathology with advanced molecular techniques to accurately characterise undifferentiated ovarian tumours. Despite aggressive multimodality therapy, outcomes remain poor, and early relapse is common, reflecting the tumour's intrinsic aggressiveness. Increasing documentation of such complex cases is essential to advancing collective knowledge, refining diagnostic algorithms, guiding future therapeutic development, and ultimately improving survival in this devastating disease.

## Author Contributions

**Mahendra Kumar:** conceptualization, methodology, data curation, writing – review and editing, writing – original draft, formal analysis, software, validation. **Dimpy Begum:** conceptualization, supervision. **Lopamundra Kakoti:** investigation, data curation, writing – review and editing, formal analysis, validation. **Bharati Seth:** data curation. **Debabrata Barmon:** conceptualization, methodology, supervision, writing – review and editing, project administration, validation. **Deepali Mittal:** data curation. **Upasana Baruah:** conceptualization, supervision, formal analysis. **Karthik Chandra Bassetty:** writing – review and editing. **P. S. Roy:** investigation.

## Funding

The authors have nothing to report.

## Ethics Statement

All procedures performed in this study adhered to the ethical standards outlined in the 1964 Helsinki Declaration and its subsequent amendments or to comparable ethical standards. The Institutional Ethics Committee of the Dr. Bhubaneswar Borooah Cancer Institute, Guwahati, Assam, approved the research protocol.

## Consent

The patient, unfortunately, succumbed to disease progression during follow‐up. Written informed consent for publication was subsequently obtained from the patient's legally authorised next of kin (her husband). Consent specifically covered publication of anonymised clinical data, treatment details, histopathological images, molecular findings, and radiological figures. All potentially identifiable information has been removed, and every reasonable effort has been undertaken to preserve patient confidentiality. The signed consent document is retained by the authors and can be provided to the journal upon request.

## Conflicts of Interest

The authors declare no conflicts of interest.

## Data Availability

The data that support the findings of this study are available on request from the corresponding author. The data are not publicly available due to privacy or ethical restrictions.
